# 

**Published:** 1889-11

**Authors:** 


### CONTENTS:

Article I.—Reflex Neurosis in Relation to Dental Pathology. By George B. Hayes, D.D.S. - - - 289
" II.—The Importance of a Thorough Mechanical Training. By David Hepburn, L. D. S. - - - 299
" III.—Neglected Advantages. By Albert H. Brockway, M.D.S. 303
" IV.—Notes of a Case of Sclerotitis Apparently of Dental Origin. By John Hern, M.D.,M.R.C.S - 309
" V.—Fatal Case of Nitrous Oxide Anæsthesia. - - 312
" VI.—Filling Root Canals of Human Teeth. By Isaac Douglass. - - - - 314
" VII.—How are Deciduous Teeth Cast Off. By Henry S. Chase, M.D - - - 321
" VIII.—Make the Foundations Firm. By Dr. G. W. Decamp. 325

### EDITORIAL.

The History of the University of Maryland Dental Department. 327
A Tragic Event Recalled. - - - - - - 329

### OBITUARY.

Elisha Parsons, M.D.,D.D.S. - - - - - - 330

### MONTHLY SUMMARY.

A Suggestive Case. - - - - - - - - 332

An Improved Way of Using the Hypodermic Method. - - 334

The Mortality Among Nurses. - - - - - - - 335

The Cocoanut as a Tæniacide. - - - - - 336

Beer Compared With Other Alcoholics. - - - - 336

